# Clarithromycin Enhances the Antibacterial Activity and Wound Healing Capacity in Type 2 Diabetes Mellitus by Increasing LL-37 Load on Neutrophil Extracellular Traps

**DOI:** 10.3389/fimmu.2018.02064

**Published:** 2018-09-10

**Authors:** Athanasios Arampatzioglou, Dimitrios Papazoglou, Theocharis Konstantinidis, Akrivi Chrysanthopoulou, Alexandros Mitsios, Iliana Angelidou, Ioanna Maroulakou, Konstantinos Ritis, Panagiotis Skendros

**Affiliations:** ^1^Laboratory of Molecular Hematology, Democritus University of Thrace, Alexandroupolis, Greece; ^2^Diabetes Clinic, Second Department of Internal Medicine, University Hospital of Alexandroupolis, Alexandroupolis, Greece; ^3^Laboratory of Cancer Genetics, Department of Molecular Biology and Genetics, Democritus University of Thrace, Alexandroupolis, Greece; ^4^First Department of Internal Medicine, University Hospital of Alexandroupolis, Alexandroupolis, Greece

**Keywords:** type 2 diabetes mellitus, neutrophil extracellular traps, LL-37, clarithromycin, diabetic infections, wound healing

## Abstract

**Background:** Type 2 diabetes mellitus (T2D) is characterized by susceptibility to bacterial infections and impaired wound healing. Neutrophil extracellular traps (NETs) and the cathelicidin antimicrobial peptide LL-37 have been implicated both in defense against bacterial infections and in wound healing process. Recently, it was shown that macrolide antibiotic clarithromycin induces the release of LL-37-bearing NETs. In T2D there has not been identified any link between NETs and LL-37 and the effect of clarithromycin in neutrophils/NETs is unknown yet.

**Methods:** Peripheral blood neutrophils were obtained from treatment-naive hyperglycemic T2D patients (naive), normoglycemic T2D patients under antidiabetic treatment (well-controlled) and healthy donors (controls). NET release and NET proteins were studied. Co-culture systems of NET structures with *E. coli* NCTC 9001 and primary skin fibroblasts were deployed to examine the *in vitro* antibacterial and fibrotic NET properties, respectively. The effect of clarithromycin was also investigated. Analysis was performed using immunofluorescence confocal microscopy, myeloperoxidase-DNA complex and LL-37 ELISA, immunoblotting and qRT-PCR.

**Results:** NETs were characterized by the presence of LL-37, however they lacked antibacterial activity, in both groups of T2D patients. Clarithromycin significantly increased the externalization of LL-37 on NETs generated from well-controlled T2D neutrophils, thus restoring NET antibacterial capacity and promoting the wound healing process via fibroblast activation and differentiation.

**Conclusion:** This study suggests that clarithromycin may add further advantage to well-controlled T2D patients, by enhancing their antibacterial defense and improving wound healing capacity of fibroblasts, through upregulation of LL-37 on NET structures.

## Introduction

Type 2 diabetes mellitus (T2D) is a metabolic disease characterized by chronic inflammation and insulin resistance, which lead to impaired glucose tolerance (1, 2). T2D represents a significant health problem with increasing prevalence worldwide. Many lines of evidence have associated T2D with increased risk for infections and impaired wound healing, features that commonly lead to serious complications, such as diabetic foot ulcers, increased hospitalization rate and high mortality (3, 4). Therefore, the development of novel therapeutic approaches against infections and wound healing incapability in T2D patients is urgently needed today.

Neutrophil extracellular traps (NETs) are a key defensive mechanism of neutrophils, an essential part of the innate immune system (5). Besides their role against infections, NETs are involved in various non-infectious diseases (6–13), including T2D (14–16) and fibrosis (17). Recently, was suggested that the pathogenic role of NETs in several diseases is defined mainly by their disease-related protein load (7, 8, 18). T2D patients are characterized by increased spontaneous NET release, independently of their glycemic status (15, 19, 20). Experimental and clinical data have shown that T2D impairs NET formation, triggering the delayed release of short-lived and unstable NETs (16, 21). This impairment has been implicated in increased susceptibility to bacterial infections (16) and wound healing incapability (14, 21). However, the mechanism through which NETs affect antimicrobial defense and wound healing process in T2D, as well as their protein load, are still largely unknown.

LL-37, the active form of human cathelicidin, is an antimicrobial peptide with an important role in defense against bacterial and fungal infections (22). It is expressed by various cell types, including neutrophils (23). LL-37-DNA complexes have been implicated in the pathogenesis of autoimmune and chronic inflammatory diseases including psoriasis (24), systemic lupus erythematosus (7), and atherosclerosis (25). Recently, our group demonstrated that the macrolide antibiotic clarithromycin induces the formation of LL-37-DNA complexes, in particular LL-37-bearing NETs, which possess potent antimicrobial activity (26). Though, whether this immunomodulatory role of such a widely used antibiotic could be utilized against susceptibility to infections in T2D is unknown. In addition, several *in vitro* and *in vivo* studies suggest that LL-37 is also associated with wound healing by promoting re-epithelialization and vascularization (27–29). However, whether LL-37 could be beneficial against impaired wound healing in T2D is still under investigation.

In this study we demonstrate for the first time that NETs in T2D are decorated with LL-37, although they are deprived of antibacterial effect. We also provide novel evidence that clarithromycin significantly improves LL-37-mediated antibacterial activity of neutrophils and healing capacity of skin fibroblasts in well-controlled T2D, by further increasing the levels of LL-37 on NETs.

## Materials and methods

### Patients and sample collection

Peripheral blood neutrophils and sera were obtained from 6 treatment-naive T2D patients with impaired glycemic control (naive T2D), 6 patients with good glycemic control (HBA1c < 7.0%) under treatment with metformin (*n* = 2) or metformin plus basal insulin (*n* = 4; well-controlled T2D), as well as 6 age- and sex-matched healthy individuals who were used as controls (control individuals). Patients were diagnosed according to the American Diabetes Association criteria (30). Patients suffering from inflammatory/autoimmune, infectious or neoplastic diseases were excluded. Patient and healthy individual characteristics are demonstrated in Table 1.

**Table 1:** Clinical characteristics of the individuals that participated in the study.

**Variable**	**Treatment-naive T2D patients**	**Well-controlled T2D patients**	**Healthy individuals**
Number (*n*)	6	6	6
Sex	3M/3F	4M/2F	3M/3F
Age (years, mean ± SD)	55.2 ± 9.1	56.5 ± 9.8	52.5 ± 8.6
FPG (mg/dl, mean ± SD)	219.3 ± 36.2	121.2 ± 20.1	90.5 ± 10.9
HbA1c (%, mean ± SD)	8.7 ± 1.2	6.3 ± 0.4	5.3 ± 0.3
BMI (kg/m^2^, mean ± SD)	31.6 ± 3.9	32.9 ± 3.6	28.1 ± 3.2
Smoking (*n*)	3	2	2
Hypertension (*n*)	5	6	0
Dyslipidemia (*n*)	4	3	0
CAD (*n*)	2	2	0
CKD (*n*)	1	2	0

*M, male; F, female; SD, standard deviation; FPG, fasting plasma glucose; HbA1c, hemoglobin A1c; BMI, body mass index; CAD, coronary artery disease; CKD, chronic kidney disease*.

Written informed consent was obtained by all individuals involved in this study. The study protocol design was in accordance with the Declaration of Helsinki and was approved by the Ethics Review Board of the University Hospital of Alexandroupolis (approval protocol number: 330/18-04-2016).

### Neutrophil isolation and stimulation studies

Blood samples were used for isolation of neutrophils within 15–30 min after sampling. Neutrophils were isolated from heparinized with Sodium heparin venous blood by Histopaque double-gradient density centrifugation (31). Cell purity was ≥98%. Isolated cells were washed once with phosphate buffered saline (PBS, 1x) before culturing. Neutrophils (1.5 × 10^6^) were cultured in 5% CO_2_ at 37°C for 210 min, in a total volume of 500 μl of Roswell Park Memorial Institute (RPMI) medium (ThermoFisher SCIENTIFIC; 11875-093) in the presence of 2% heterologous healthy donor serum. For *in vitro* stimulation with clarithromycin, neutrophils were treated with clarithromycin (Anfarm Hellas; 50 mg/ml) at a clinically relevant final concentration of 2 μg/ml for 210 min. Preparation of cigarette smoke extract (CSE) and treatment of neutrophils with CSE were performed as previously described (17).

### Immunofluorescence

Prior to incubation or stimulation, isolated neutrophils were seeded in lysine-coated glass coverslips (Neuvitro; H-12-1.5-pdl). Fixation was performed using 4% paraformaldehyde (Scharlab; FO00102500) for 60 min at room temperature. Non-specific binding sites were blocked with 5% goat serum (Invitrogen; PCN5000) in PBS 1x. Samples were stained using mouse monoclonal anti-LL-37 antibody (Santa Cruz; sc-166770; 1:200) or mouse monoclonal anti-IL-17 antibody (R&D SYSTEMS; MAB3171; 1:200) and rabbit polyclonal anti-NE antibody (Santa Cruz; sc-25621; 1:200). Following three washes with PBS 1x, a polyclonal goat anti-rabbit Alexa Fluor 647 antibody (Invitrogen; A-21244; 1:500) was utilized as secondary antibody for rabbit polyclonal anti-NE. Following three washes with PBS 1x, a polyclonal rabbit anti-mouse Alexa Fluor 488 antibody (Invitrogen; A-11059; 1:500) was utilized as secondary antibody for mouse monoclonal anti-LL-37 or mouse monoclonal anti-IL-17. DAPI (Sigma-Aldrich; D9542; 1:) was used for DNA counterstaining at a final concentration of 1 μg/ml. Visualization was performed in a confocal microscope (Revolution spinning disk confocal system; Andor, Ireland) with PLAPON 60xO/TIRFM-SP [numerical aperture [NA], 1.45] objective (Olympus).

### NET isolation

A total of 1.5 × 10^6^ untreated or stimulated with clarithromycin neutrophils were cultured in 5% CO_2_ at 37°C for 210 min, in a total volume of 1200 μl of RPMI medium. After medium removal, cells were washed twice with pre-warmed RPMI medium. NET structures were collected on supernatant medium after vigorous agitation of the culture plate (32). Application of mechanical forces to the culture plate by shaking it vigorously leads to the detachment of formed NET structures from attached cells to the culture plastic, resulting in their release into the supernatant medium. The medium was centrifuged at 20 × g for 5 min. The supernatant phase containing NET structures was collected and stored at −20°C.

### NET protein purification

NET protein purification was performed as previously described (12). In brief, neutrophils (1.5 × 10^6^) were cultured as described in the section NET isolation. After medium removal, cells were washed twice with pre-warmed RPMI medium and incubated at 37°C for 10 min. Formed NET structures were digested with 10 U/ml DNase-1 (Fermentas, 798 Cromwell Park, USA) in 1 ml RPMI at 37°C for 20 min. DNase-1 activity was blocked with 5 mM ethylenediaminetetraacetic acid (EDTA; Applichem, GmbH, Stockholm, Sweden). Supernatant medium containing digested NETs was centrifuged at 300 × g for 15 min to remove whole cells and at 16000 × g for 10 min to remove cellular debris. The supernatant phase was collected and stored overnight at-20°C, after the triple amount of acetone was added. The solution was centrifuged at 16000 × g for 15 min. The supernatant phase was removed. Pellet containing purified NET proteins was lysed in lysis buffer [1% Triton X-100 and 150 mM NaCl in 20 mM HEPES [pH 7.5]] with protease inhibitors (Complete Protease Inhibitor Tablets; Roche) and Stored at −20°C.

### Bacterial strains

Bacterial strain, *E. coli* NCTC 9001, were cultured as previously described (33). Bacteria were preserved in glycerol broth at −80°C. After overnight culture on MacConkey agar, bacteria were suspended in saline to an optical density of 0.5 McFarland, corresponding to a concentration of ~10^8^ CFU/ml. *E. coli* NCTC 9001 were cocultured with NET structures isolated as described in the section NET isolation. For LL-37 inhibition on LL-37-bearing NETs, they were incubated with anti-LL-37 antibody at a final concentration of 5 μg/ml prior to their introduction to bacterial cultures, as previously described (26). Mouse monoclonal IgG1 antibody was used as a control to anti-LL-37 and did not affect NET-induced bacterial killing.

### MPO-DNA complex ELISA

To quantify NET release by untreated or stimulated with clarithromycin neutrophils, MPO-DNA complex was measured in NETs isolated from 1.5 × 10^6^ neutrophils as previously described (26). In brief, an F-bottom high-binding 96-well-plate was coated with human anti-MPO ab (1/500 dilution; Hycult Biotech, HM2164) overnight at 4°C. After three washes with PBS 1x, isolated NET structures were added to the wells. Anti-double strand DNA ab (1/25 dilution; Sigma-Aldrich, 11774425001) was used as a detection antibody. The plate was incubated for 2 h with shaking at room temperature. After three washes with incubation buffer, peroxidase substrate [2,2′-azinobis[3-ethylbenzthiazolinesulfonic acid; ABTS]] was added to the wells. After 10 min of incubation at room temperature in the dark, absorbance was measured at 405 nm. NET release was calculated as percent increase compared to that in control NETs.

### Western blot analysis

Western blot analysis for the measurement of LL-37 and IL-17 levels on NETs was performed as previously described (26) Briefly, purification of NET proteins was conducted as described in the section NET protein purification. Polyvinylidene difluoride (PVDF) membranes were incubated with mouse monoclonal anti-LL-37 antibody (Santa Cruz; sc-166770; 1:500) overnight at 4°C. Membranes were probed with horseradish peroxidase (HRP)-conjugated secondary antibody (stock concentration, 400 μg/ml; 1:1500) for 45 min at room temperature. Moreover, mouse monoclonal anti-IL-17 antibody (R&D SYSTEMS; MAB3171; 1:250) was used for the measurement of IL-17 levels on NETs, using the same protocol.

### LL-37 ELISA

LL-37 levels on isolated NET structures were quantified using an LL-37 ELISA kit according to the instructions of the manufacturer (Hycult Biotech, Uden, NL). In brief, isolated NET structures were added to the wells of the ELISA kit microwell strips. After incubation for 1 h at room temperature, the plate was washed four times with wash/dilution buffer. Tracer was added to each well. After incubation for 1 h at room temperature, the wash procedure was repeated. Streptavidin-peroxidase was added to each well. After incubation for 1 h at room temperature, the wash procedure was repeated. TMB substrate was added to each well. After incubation for 30 min at room temperature, stop solution was added to each well. Absorbance was measured at 450 nm. LL-37 levels on NET structures were calculated in ng/ml.

### Scratch wound healing assay

The migratory capacity of SFs was evaluated using a cell migration assay according to the instructions of the manufacturer (Cell Biolabs), as previously described (34). Primary SFs were transferred to a 24-well plate (Corning Incorporated, New York, USA; 0.3–0.5 × 10^6^ cells/well), after an insert was placed in each well. Cells were incubated in complete Dulbecco's modified Eagle's medium (DMEM; Gibco BRL, New York, USA) for 24 h at 37°C. Inserts were removed to generate a 0.9-mm “wound field.” Medium was also removed, and cells were cultured in low-serum DMEM for 2 h at 37°C. During stimulation, SFs were cultured in low-serum DMEM in the presence of 10% isolated NET structures for 24 h at 37°C. Cells were stained with May Grünwald-Giemsa and visualized by reverse light microscope (Axiovert 25; Zeiss). The migration rate was measured according to the instructions of the manufacturer. LL-37 inhibition on LL-37-bearing NETs was performed as described in the section Bacterial Strains.

### RNA isolation, cDNA synthesis, and qRT-PCR

RNA isolation and cDNA synthesis were conducted in SFs as previously described (35). Real-time PCR for alpha-actin-2 (ACTA2) and CCN2 was performed in SFs after 24 h and 48 h of stimulation respectively, based on optimization experiments. In brief, real-time PCR was performed using SYBR Green qPCR Master Mix (2x) gene expression master mix (Fermentas, St. Leon-Rot, Germany) on a Chromo4TMReal-Time Detector (Bio-Rad, CA, USA). GAPDH was used as the house-keeping gene to normalize the expression levels of target genes. The following oligonucleotide primers, designed by Beacon Designer™ 4.0, were used: *ACTA2* (forward: 5′- ACG CAC AAC TGG CAT CG−3′, reverse: 5′- CGG ACA ATC TCA CGC TCA G−3′), *CCN2* (forward: 5′- ACC AAT GAC AAC GCC TCC TG−3′, reverse: 5'- TTG CCC TTC TTA ATG TTC TCT TCC−3′), *GAPDH* (forward: 5′- GGG AAG CTT GTC ATC AAT GG−3′, reverse: 5'- CAT CGC CCC ACT TGA TTT TG−3′). The PCR protocol used included the following steps: 52°C for 5 min; 95°C for 2 min; 35 cycles of: 95°C for 15 s and 51°C for 40 s; 52°C for 5 min; melting curve analysis.

### Collagen measurement

The soluble collagen types (I–V) were determined using a Sircol Collagen Assay Kit according to the instructions of the manufacturer (Biocolor), as previously described (34). Briefly, primary SFs were transferred to a 6-well plate (Corning Incorporated, New York, USA) and cultured in complete DMEM for 72 h at 37°C. Medium was removed and cells were cultured in low-serum DMEM for 24 h at 37°C. During stimulation, SFs were cultured in low-serum DMEM in the presence of 10% isolated NET structures for 48 h at 37°C. Culture supernatant was collected and centrifuged at 20 × g for 5 min. Contained collagen was measured after overnight treatment with the manufacturer's isolation and concentration reagent. In this set of studies, collagen production was measured in culture supernatants after 48 h of stimulation, based on optimization experiments.

### Statistical analysis

Statistical analyses were performed using Kruskal-Wallis test, followed by Dunn's test for multiple comparisons. The overall *p*-value for the Kruskal-Wallis (KW) test for each graph is indicated in the corresponding figure legend. *P*-values less or equal to 0.05 were considered significant. All statistical analyses were performed using GraphPad Prism 6.

## Results

### NETs in T2D are decorated with LL-37

Considering that LL-37 has both antimicrobial and wound healing properties (28) and is released through NETs under certain inflammatory conditions (26), as well as that T2D is characterized by chronic inflammation (15) and spontaneous NETosis (19), we investigated whether LL-37 is present on diabetic NET structures.

**Figure 1:**
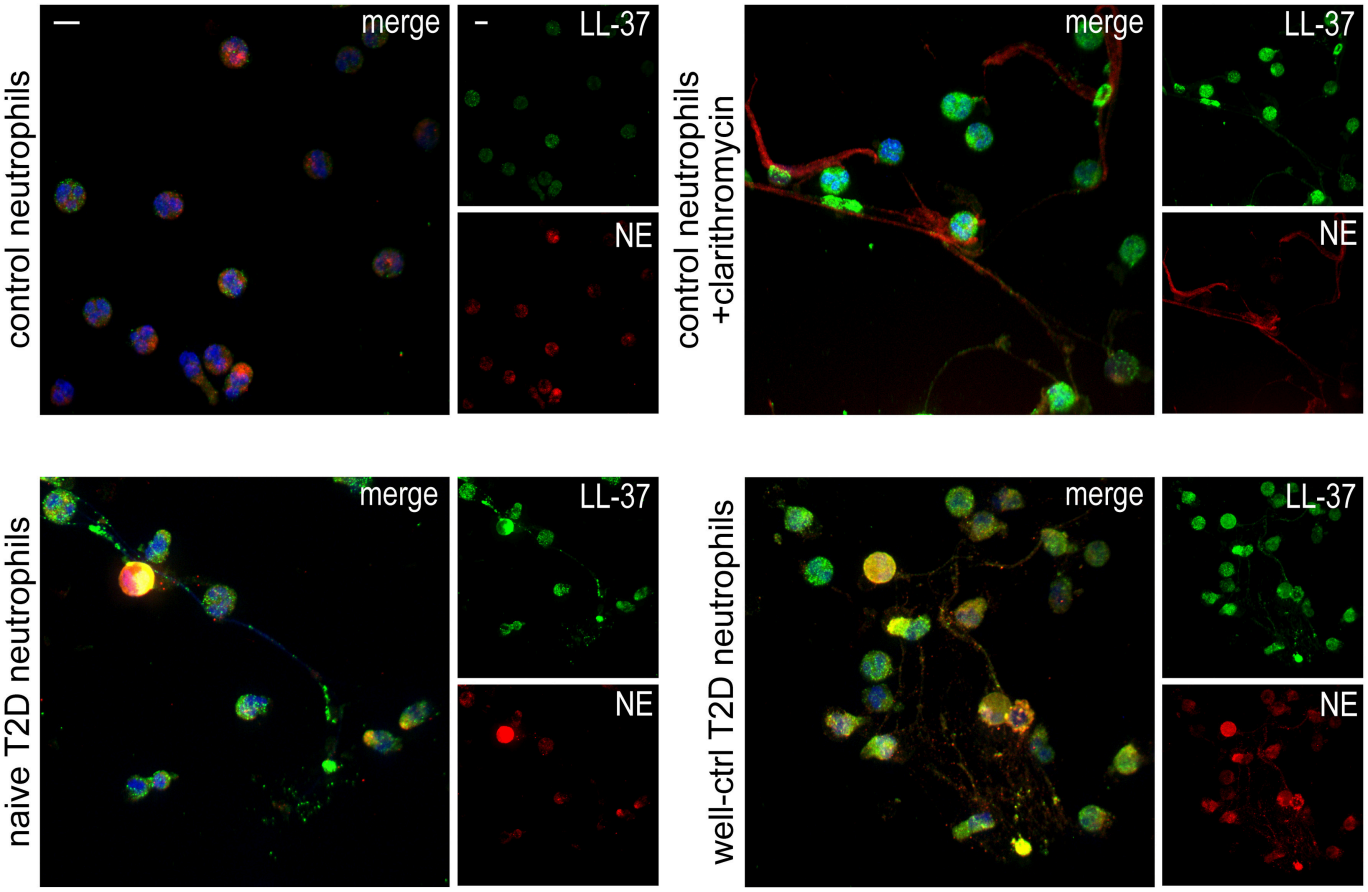
T2D neutrophils release LL-37-bearing NETs. Confocal microscopy for LL-37/NE staining in neutrophils isolated from treatment-naive and well-controlled T2D patients. Neutrophils isolated from healthy individuals treated with clarithromycin or not were used as positive and negative controls, respectively. Blue: DAPI, Green: LL-37, Red: NE. One representative out of six independent experiments is shown. Original magnification: x600, Scale bar−5μm.

Neutrophils isolated from both treatment-naive and well-controlled T2D patients released LL-37-bearing NETs, as assessed by immunofluorescence (Figure 1). NETs generated by neutrophils derived from healthy individuals upon *in vitro* stimulation with clarithromycin were used as positive control (26). These data demonstrate that neutrophils of naive and well-controlled T2D patients spontaneously release NETs carrying LL-37.

### Clarithromycin restores the *in vitro* antibacterial activity of NETs in well-controlled T2D by increasing NET-bound LL-37

Based on the above finding, we investigated the antimicrobial activity of LL-37-bearing diabetic NET structures.

**Figure 2:**
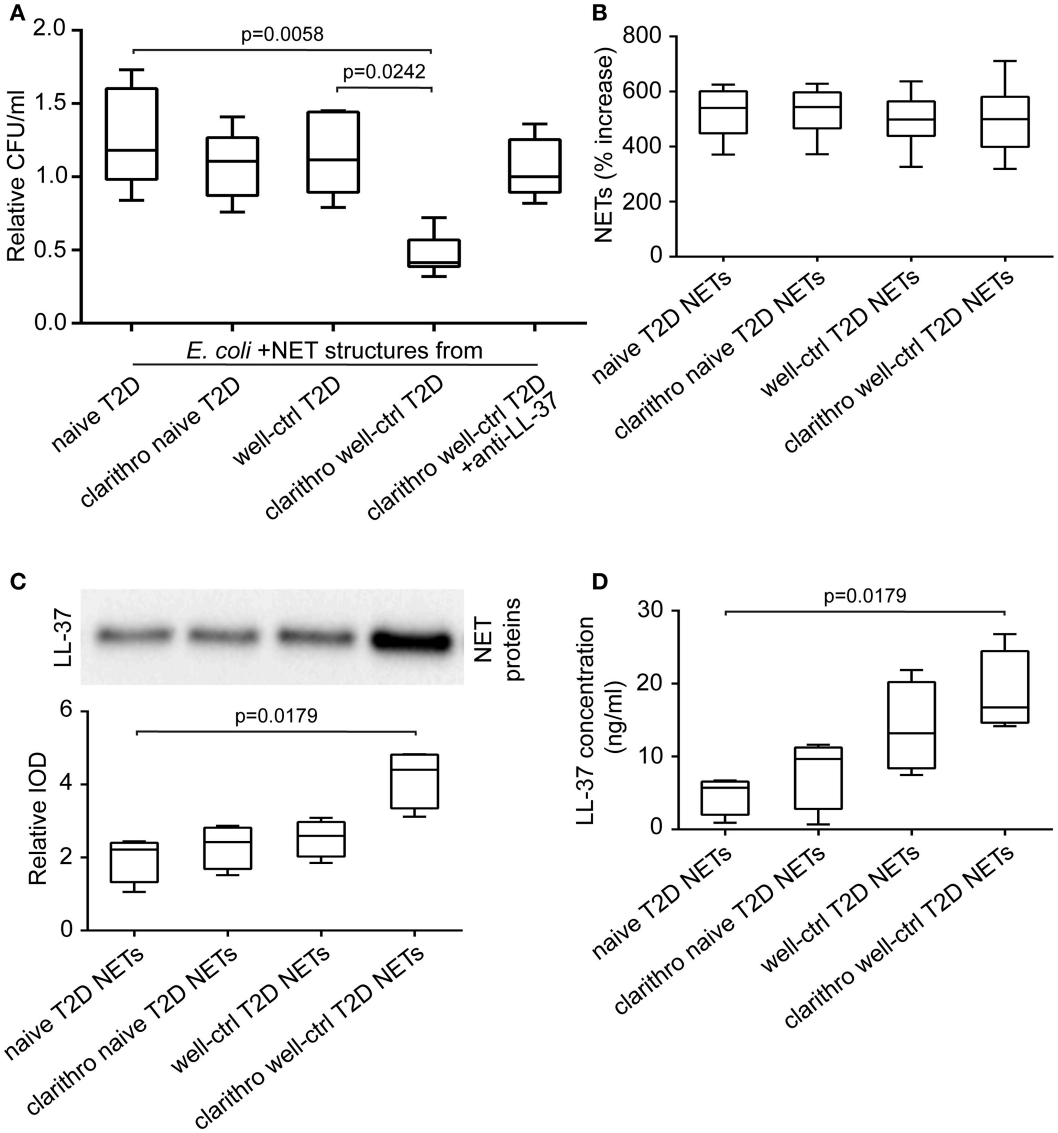
Clarithromycin restores the antibacterial properties of NETs derived from well-controlled T2D patients, by increasing NET-bound LL-37 levels. **(A)** Cultures of *E. coli* NCTC 9001 in the presence of NETs from treatment-naive and well-controlled T2D patients *in vitro* generated spontaneously or using clarithromycin. Anti-LL-37 antibody was used as an inhibitor of NET-bound LL-37. Untreated *E. coli* and NETs from healthy individuals *in vitro* generated spontaneously or using clarithromycin were used as controls. Relative CFU was calculated as ratio to untreated *E. coli* value. Overall KW *p*-value was 0.0051. **(B)** MPO-DNA complex levels in NET structures from healthy individuals, naive and well-controlled T2D patients generated *in vitro* spontaneously or using clarithromycin, as assessed by ELISA. NET release was calculated as percent increase compared to control NETs value. Overall KW *p*-value was 0.7982. **(C)** LL-37 in purified NET proteins isolated from NETs described in panel B. Relative IOD was calculated compared to control NETs value. Overall KW *p*-value was 0.0070. **(D)** LL-37 levels on NET structures described in panel B, as assessed by ELISA. Overall KW *p*-value was 0.0036. **(A,B)** Data from six or **(C,D)** four independent experiments presented in box-and-whiskers plots including minimum and maximum values as well as median and IQR. CFU—colony forming units. IOD—integrated optical density.

*E. coli* NCTC 9001 were cultured in the presence or absence of NETs released from neutrophils isolated from naive and well-controlled T2D patients (naive and well-ctrl T2D NETs, respectively). Diabetic NETs had no inhibitory effect on bacterial growth, irrespective of whether they derived from treatment-naive or well-controlled patient's neutrophils (Figure 2A). These data indicate that diabetic NETs, although decorated with the potent antimicrobial peptide LL-37, are characterized by incapability to inhibit bacterial growth *in vitro*.

We next examined whether clarithromycin, a known inducer of LL-37-bearing NETs (26), could induce the *in vitro* antibacterial activity of diabetic NET structures.

*E. coli* NCTC 9001 cultures were performed in the presence or absence of NETs generated by neutrophils derived from naive and well-controlled T2D patients upon *in vitro* stimulation with clarithromycin. Clarithromycin-induced NETs from neutrophils of well-controlled patients (clarithro-well-ctrl T2D NETs) significantly reduced bacterial growth compared to both naive (*p* = 0.0058) and well-ctrl T2D NETs (*p* = 0.0242; Figure 2A). On the contrary, clarithromycin-induced NETs from neutrophils of naive patients (clarithro-naive T2D NETs) had no inhibitory effect on E. coli growth (Figure 2A). When LL-37 on clarithro-well-ctrl T2D NETs was neutralized with anti-LL-37 antibody, their antibacterial properties were significantly abrogated (Figure 2A). Treatment with monoclonal IgG1 antibody had no effect. These findings suggest that clarithromycin restores the *in vitro* antibacterial properties of NET structures in normoglycemic T2D patients in an LL-37-dependent manner.

Stimulation with clarithromycin did not further enhance the ability of diabetic neutrophils to release NETs as assessed by MPO-DNA complex ELISA (Figure 2B). However, the levels of LL-37 on clarithro-well-ctrl T2D NETs were significantly increased compared to naive T2D NETs, as assessed by both immunoblotting of purified NET proteins (*p* = 0.0179; Figure 2C) and quantification of LL-37 on NET structures using ELISA (*p* = 0.0179; Figure 2D). Thus, the *in vitro* antibacterial activity of clarithro-well-ctrl T2D NETs may be attributed to the increased externalization of LL-37 on these NETs.

### Clarithromycin induces NETs with a beneficial role in wound healing in T2D

Taking into account the abovementioned data together with the increasing evidence that LL-37 promotes wound healing (27, 29), we sought to investigate whether LL-37-bearing diabetic NETs are implicated in wound healing process, as well as the effect of clarithromycin.

**Figure 3:**
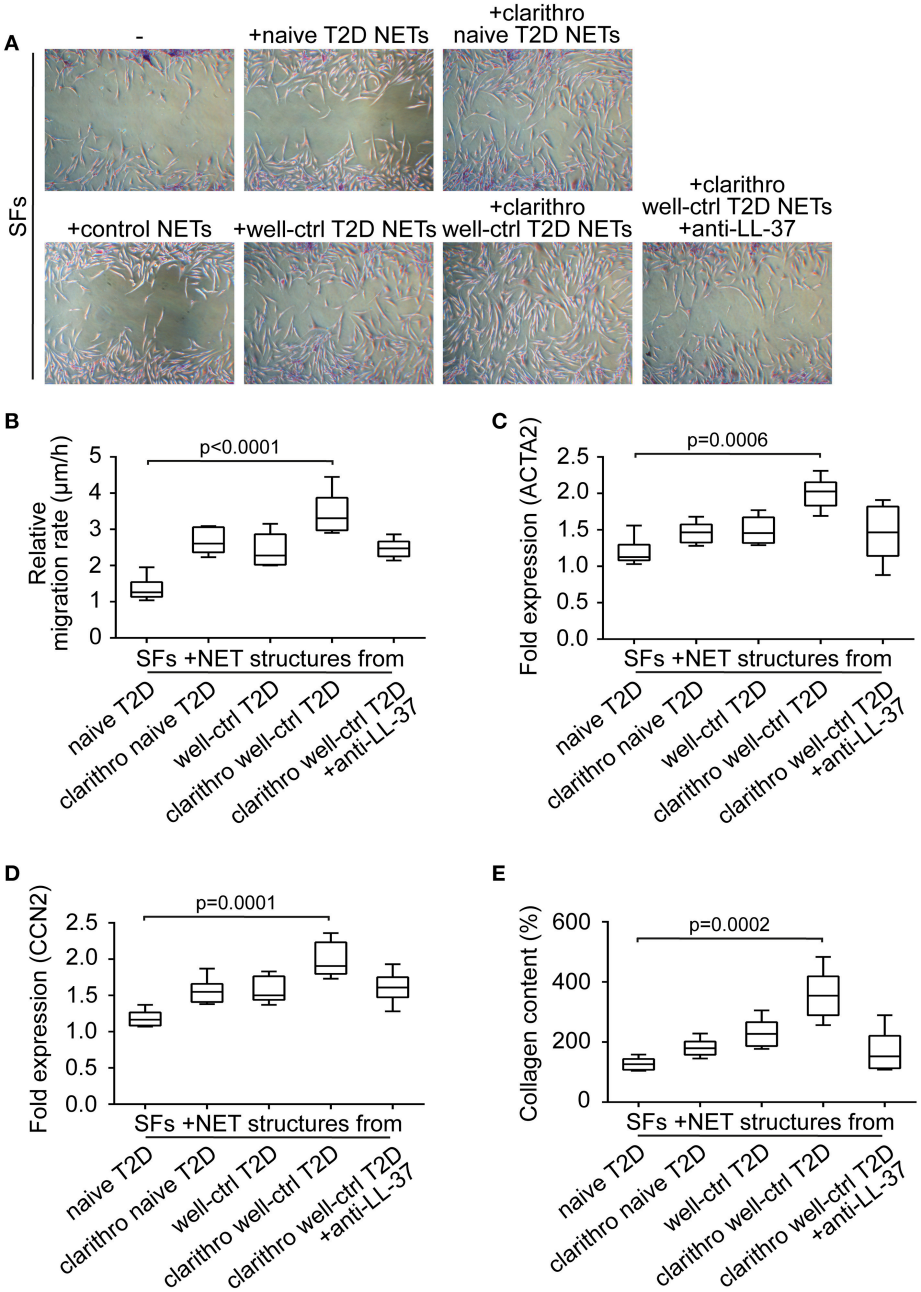
Clarithromycin-induced NETs from neutrophils of well-controlled T2D patients promote activation and differentiation of SFs. **(A)** Scratch wound healing assay, **(B)** migration rate, **(C)** ACTA2 and **(D)** CCN2 mRNA expression, and **(E)** collagen production in SFs treated with NET structures generated *in vitro* spontaneously or using clarithromycin. Anti-LL-37 antibody was used as an inhibitor of NET-bound LL-37. Untreated SFs and NETs from healthy individuals generated *in vitro* spontaneously were used as controls. **(A)** One representative out of six independent experiments is shown. Original magnification: x50. **(B)** Relative migration rate, **(C,D)** mRNA expression and **(E)** collagen content were calculated as ratio, fold increase and percent increase respectively compared to untreated SFs value. **(B–E)** Data from six independent experiments presented in box-and-whiskers plots including minimum and maximum values as well as median and IQR. Overall KW *p*-value was **(B)** 0.0003, **(C)** 0.0026, **(D)** 0.0006, and **(E)** 0.0003.

To examine the involvement of diabetic NETs in wound healing, we studied their effect on the activation and differentiation into myofibroblasts of human skin fibroblasts (SFs) (17). Primary SFs were incubated with naive and well-ctrl T2D NETs, as well as clarithro-naive and clarithro-well-ctrl T2D NETs. Clarithro-well-ctrl T2D NETs significantly enhanced migration rates compared to naive T2D NETs (*p* < 0.0001), as assessed by scratch wound healing assay (Figures 3A,B). Moreover, they upregulated a-SMA (ACTA2; *p* = 0.0006; Figure 3C), and CCN2 (*p* = 0.0001; Figure 3D) mRNA levels along with collagen production (*p* = 0.0002; Figure 3E) that indicate activation of SFs and their differentiation to myofibroblasts, respectively. Well-ctrl T2D NETs and clarithro-naive T2D NETs also demonstrated a moderate, though non-significant, enhancing effect on the activation and differentiation of SFs. The significant inductive impact of clarithro-well-ctrl T2D NETs on the activation and differentiation of SFs was attributed to the increased levels of LL-37 on these NETs (Figure 2C), since this impact was abrogated when LL-37 was neutralized with anti-LL-37 antibody (Figures 3A–E), whereas monoclonal IgG1 antibody had no effect. These findings indicate that upregulation of LL-37 on T2D NETs by clarithromycin is able to promote activation and differentiation of SFs.

Considering previous studies demonstrating that IL-17 on NETs also promotes fibrotic activity of fibroblasts (17), we examined whether neutrophils from T2D patients express IL-17 and/or externalize it on NETs (Supplementary Figure S1). NETs generated by neutrophils derived from healthy individuals after *in vitro* stimulation with cigarette smoke extract (CSE) were used as positive control (17). Neither neutrophils from treatment-naive patients nor those from well-controlled patients released NETs decorated with IL-17 (Supplementary Figure S1). Clarithromycin did not induce IL-17 expression as well (Supplementary Figure S1).

These data further support that the increased activation and differentiation of SFs upon their stimulation with clarithro-well-ctrl T2D NETs was due to the increased levels of NET-bound LL-37.

## Discussion

Neutrophils of T2D patients are characterized by increased NET formation (14, 15, 19), however the proteins externalized on diabetic NETs and their function are still largely unknown. In this study, NETs in T2D were shown to be decorated with the cathelicidin LL-37 for the first time to our knowledge. In addition, treatment of neutrophils isolated from well-controlled T2D patients with the widely used antibiotic clarithromycin further increased the levels of LL-37 externalized on NETs, leading to significant *in vitro* induction of antibacterial activity in diabetic neutrophils and healing capacity in skin fibroblasts.

In line with previous studies associating impaired NET formation in T2D with susceptibility to bacterial infections (16), we demonstrate that NETs in T2D, though LL-37-bearing, are incapable of inhibiting bacterial growth *in vitro*. Considering that NET chromatin scaffold is crucial for NET structures and their proteins to gain function (5, 36, 37), we could hypothesize that the defective antimicrobial properties of LL-37-bearing NETs in T2D is due to a somehow unstable NET scaffold as a result of hyperglycemic environment (16).

Treatment with clarithromycin restored the defective antibacterial capacity of well-ctrl T2D NETs by upregulating LL-37 externalization on them, while had no effect on NETosis levels. Bearing in mind a recent study suggesting that LL-37 derived from LL37-DNA complexes attacks mycobacteria in macrophage phagolysosomes (38), we could assume that clarithromycin-induced NET-bound LL-37 enhances the efficiency of macrophages of T2D patients against intracellular bacteria, as well. Taken together, we propose that clarithromycin could significantly enhance the immune defense of T2D patients against bacterial infections, adding important value to good glycemic control.

Even though previous studies have shown that NETosis impairs wound healing in T2D (14, 21), herein we provide new evidence that LL-37 externalized on T2D NETs can be a potent inducer of fibrotic activity of SFs. This effect is partial in both naive and well-controlled T2D patients but increases significantly when well-ctrl T2D NETs are induced by clarithromycin. Although it has been suggested that LL-37 promotes wound healing (28), this study provides a novel mechanistic basis strongly implicating LL-37 through NETs in the activation of fibroblasts and their differentiation into collagen producing myofibroblasts. This role has also been attributed to IL-17 in other disease models (17). Thus, we suggest that treatment with clarithromycin could benefit T2D patients suffering from impaired wound healing, by modifying the LL-37-mediated function of their NETs.

**Figure 4:**
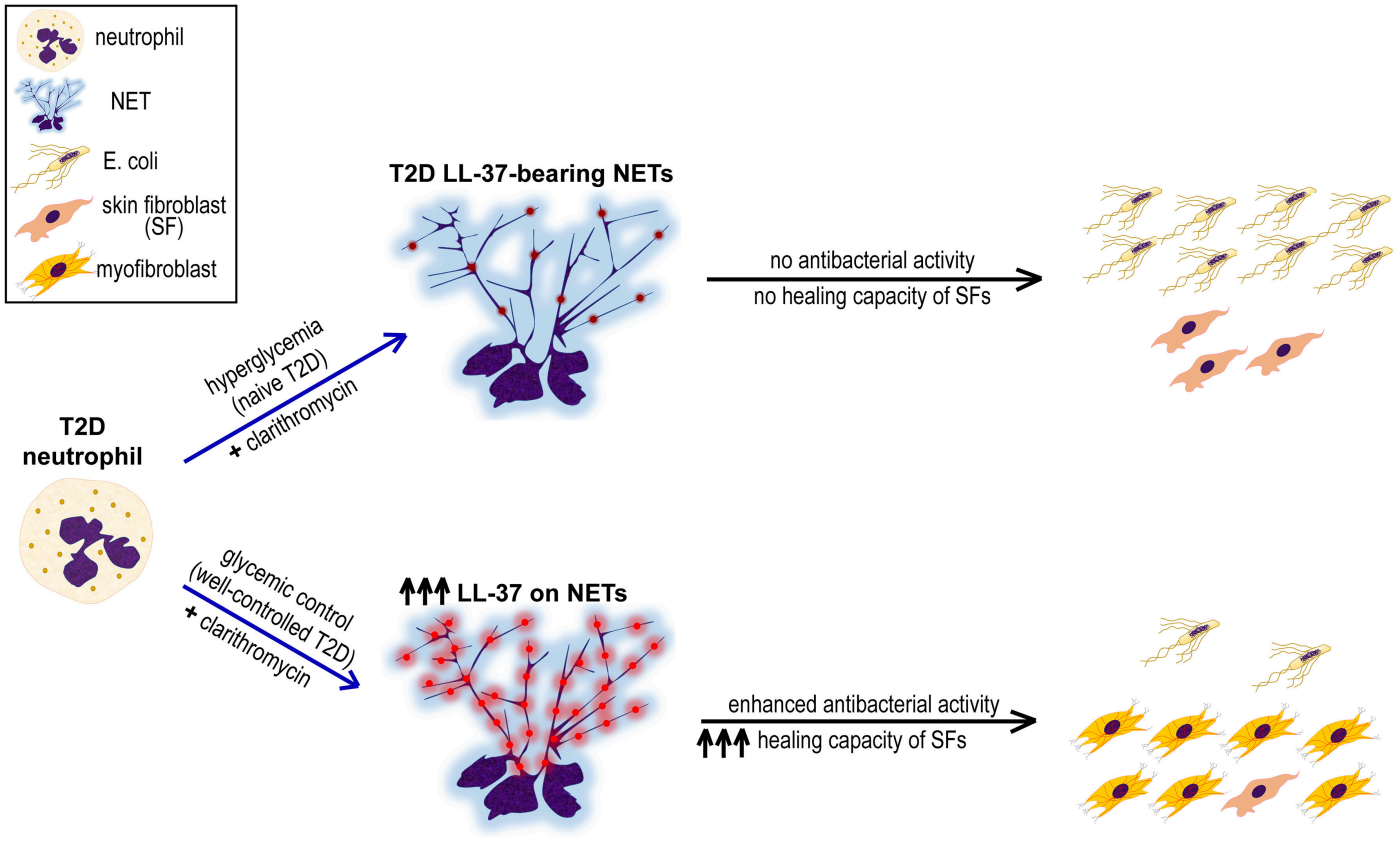
Proposed mechanism underlining the synergistic beneficial effect of good glycemic control and clarithromycin treatment on neutrophils of T2D patients. Hyperglycemic environment induces the formation of LL-37-bearing NETs from T2D neutrophils, however they neither inhibit *E. coli* growth nor promote proliferation and differentiation of skin fibroblasts (SFs). The combination of glycemic control and clarithromycin increases externalization of LL-37 (red color) on T2D NETs, restoring the antibacterial activity of neutrophils and enhancing the healing capacity of SFs.

Taken together, this study indicates the presence of LL-37, a key antimicrobial peptide known also to promote wound healing, on diabetic NET structures. *In vitro* treatment with clarithromycin in combination with proper glycemic control increase externalization of LL-37 on T2D NETs significantly, restoring antibacterial activity of neutrophils and enhancing healing capacity of SFs (Figure 4). Probably, disturbances of innate immune functions that characterize neutrophils under hyperglycemic conditions (39) circumvent efficient responses to various triggers, such as clarithromycin, and impair NET scaffold stability (16). These defects are ameliorated, at least partially, through good glycemic control. However, LL-37-bearing well-ctrl T2D NETs are still lacking efficient antibacterial and healing capacity. We propose that this is due to relatively low levels of LL-37 on well-ctrl T2D NETs since clarithromycin restores their LL-37-dependent functionality by significantly increasing externalization of NET-bound LL-37. Given that function of NET structures and their proteins is largely associated with the integrity of chromatin scaffold (5, 18), the possibility that clarithromycin also interacts directly with NET scaffold could be an interesting point for future investigation.

Despite proper glycemic control several T2D patients remain susceptible to infections and suffer from defective wound healing, which in turn increases the risk for infections (40, 41). This study suggests that immunomodulatory effects of clarithromycin may be beneficial in T2D patients by invigorating both host defense against pathogens and wound healing capability. The conduction of *in vivo* intervention experiments was beyond the scope of this study including limitations of the Ethics Review Committee approval. Beyond that, the data presented here encourage the design of preclinical and clinical trials with macrolides, or other immunomodulatory agents targeting neutrophil LL-37 without increasing the risk of antibiotic resistance, in T2D (42).

## Author contributions

AA conducted experiments, analyzed the data and wrote the manuscript. DP contributed to data analysis and wrote the manuscript; TK, AC, AM, and IA performed experiments and contributed to data analysis. IM critically revised the manuscript. KR and PS conceived and designed the study, were involved in the experimental procedure and data analysis, wrote and revised the manuscript and co-supervised the study. All authors have approved the submitted manuscript.

### Conflict of interest statement

The authors declare that the research was conducted in the absence of any commercial or financial relationships that could be construed as a potential conflict of interest.
