# Supplementary material for: Clarithromycin Enhances the Antibacterial Activity and Wound Healing Capacity in Type 2 Diabetes Mellitus by Increasing LL-37 Load on Neutrophil Extracellular Traps
Source: Front Immunol. 2018 Sep 10;9:2064. doi: 10.3389/fimmu.2018.02064 (PMC6139320; doi:10.3389/fimmu.2018.02064)
Supplement: Supplementary file 1 [file Image_1.pdf]

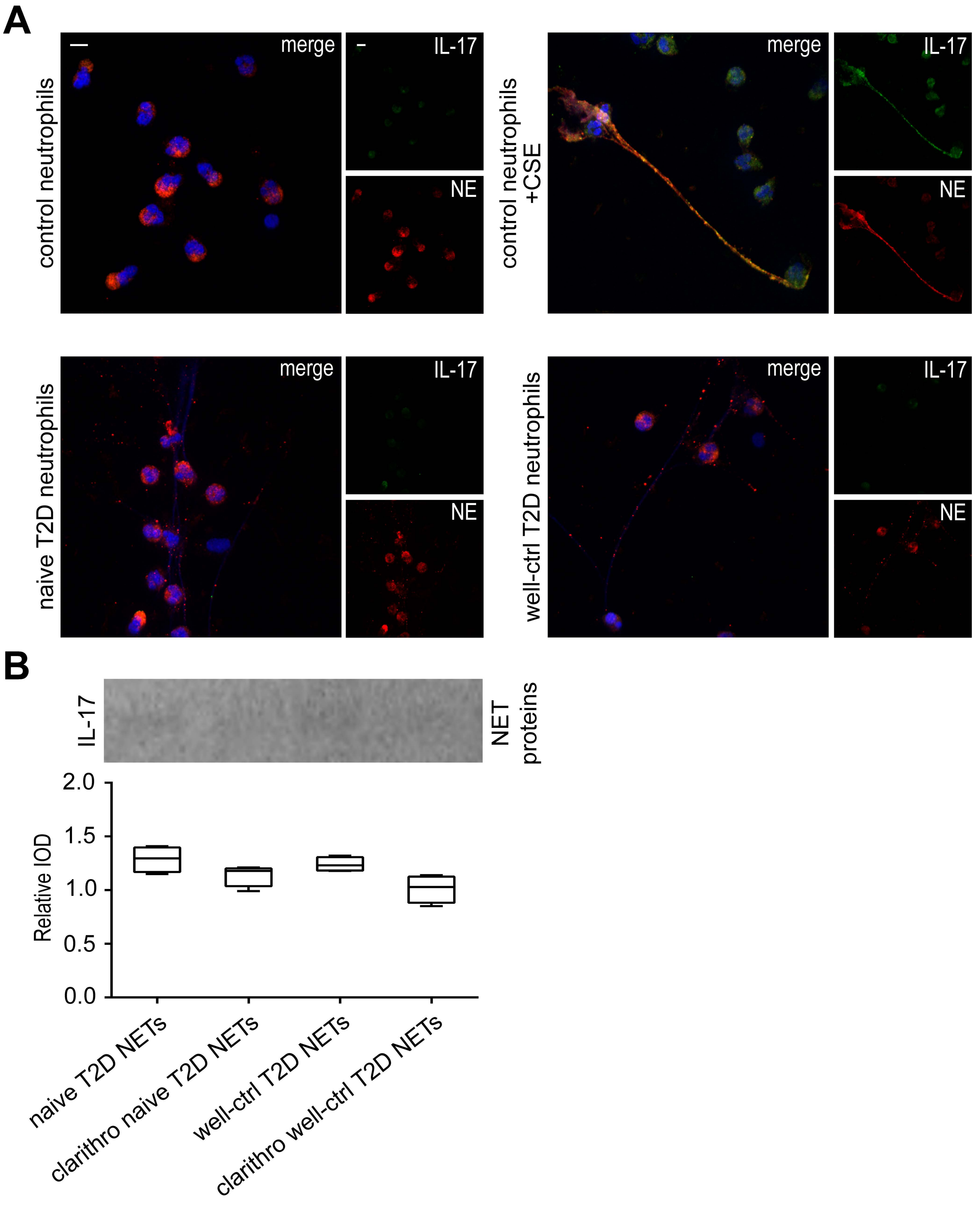

A
merge
IL-17
NE
control neutrophils
control neutrophils +CSE
naive T2D neutrophils
well-ctrl T2D neutrophils
B
IL-17
NET proteins
Relative IOD
2.0
1.5
1.0
0.5
0.0
naive T2D NETs
clarithro naive T2D NETs
well-ctrl T2D NETs
clarithro well-ctrl T2D NETs

Supplementary figure legend

Supplementary figure S1. T2D NETs are not decorated with IL-17. (A) Confocal microscopy for IL-17 / NE staining in neutrophils isolated from treatment-naive and well-controlled T2D patients. Neutrophils isolated from healthy individuals treated with cigarette smoke extract (CSE) or not were used as positive and negative controls, respectively. **(B)** IL-17 in purified NET proteins derived from NETs from naive and well-controlled T2D patients generated *in vitro* spontaneously or using clarithromycin. Relative IOD was calculated compared to control NETs value. (A) Blue: DAPI, Green: IL-17, Red: NE. One representative out of six independent experiments is shown. Original magnification: x600, Scale bar – 5μm. (B) Data from four independent experiments presented in box-and-whiskers plots including minimum and maximum values as well as median and IQR. Overall KW p-value was 0.0092. IOD – integrated optical density.
